# Cytostatic Bacterial Metabolites Interfere with 5-Fluorouracil, Doxorubicin and Paclitaxel Efficiency in 4T1 Breast Cancer Cells

**DOI:** 10.3390/molecules29133073

**Published:** 2024-06-27

**Authors:** Szandra Schwarcz, Petra Nyerges, Tímea Ingrid Bíró, Eszter Janka, Péter Bai, Edit Mikó

**Affiliations:** 1 Department of Medical Chemistry, Faculty of Medicine, University of Debrecen, 4032 Debrecen, Hungary; schwarcz.szandra@med.unideb.hu (S.S.); nyerges.petra@med.unideb.hu (P.N.); birotimeaingrid@gmail.com (T.I.B.); baip@med.unideb.hu (P.B.); 2 Department of Dermatology, MTA Centre of Excellence, Faculty of Medicine, University of Debrecen, 4032 Debrecen, Hungary; janka.eszter.a@gmail.com; 3 HUN-REN-UD Allergology Research Group, University of Debrecen, 4032 Debrecen, Hungary; 4 HUN-REN-UD Cell Biology and Signaling Research Group, University of Debrecen, 4032 Debrecen, Hungary; 5 MTA-DE Lendület Laboratory of Cellular Metabolism, 4032 Debrecen, Hungary; 6 Research Center for Molecular Medicine, Faculty of Medicine, University of Debrecen, 4032 Debrecen, Hungary

**Keywords:** 5-fluorouracil, doxorubicin, gemcitabine, irinotecan, methotrexate, rucaparib, paclitaxel, cadaverine, indolepropionic acid, indoxylsulfate, cell proliferation, breast cancer

## Abstract

The microbiome is capable of modulating the bioavailability of chemotherapy drugs, mainly due to metabolizing these agents. Multiple cytostatic bacterial metabolites were recently identified that have cytostatic effects on cancer cells. In this study, we addressed the question of whether a set of cytostatic bacterial metabolites (cadaverine, indolepropionic acid and indoxylsulfate) can interfere with the cytostatic effects of the chemotherapy agents used in the management of breast cancer (doxorubicin, gemcitabine, irinotecan, methotrexate, rucaparib, 5-fluorouracil and paclitaxel). The chemotherapy drugs were applied in a wide concentration range to which a bacterial metabolite was added in a concentration within its serum reference range, and the effects on cell proliferation were assessed. There was no interference between gemcitabine, irinotecan, methotrexate or rucaparib and the bacterial metabolites. Nevertheless, cadaverine and indolepropionic acid modulated the Hill coefficient of the inhibitory curve of doxorubicin and 5-fluorouracil. Changes to the Hill coefficient implicate alterations to the kinetics of the binding of the chemotherapy agents to their targets. These effects have an unpredictable significance from the clinical or pharmacological perspective. Importantly, indolepropionic acid decreased the IC_50_ value of paclitaxel, which is a potentially advantageous combination.

## 1. Introduction

Oncobiosis is the dysbiosis associated with neoplastic diseases. Oncobiosis is associated with numerous cancers and affects multiple microbiome compartments [1]. There are three major pathways through which the oncobiome can supports tumor progression and metastasis formation: (1) direct colonization of the tumor tissue, (2) immune suppression, and (3) the production of bacterial metabolites and toxins [2]. Although these pathways are all equally active in breast cancer, the suppression of bacterial metabolite production has key role in supporting cancer progression [2]. 

Multiple bacterial metabolites have been identified with cytostatic [2,3,4,5,6,7,8,9,10,11,12,13], pro-proliferative [14,15,16,17,18,19,20,21,22,23] or mixed [13] properties in breast cancer. These metabolites are chemically very diverse. Metabolites with cytostatic properties elicit multi-pronged effects involving the induction of an anti-Warburg-type metabolic rearrangement and the induction of mild oxidative stress, which block the epithelial–mesenchymal transition, and reduction in the proportions of cancer stem cells, culminating in cytostasis and a reduced metastatic and recurrence potential [2].

There are numerous reports showing that the microbiome interferes with the metabolism and the effectiveness of the chemotherapy agents used in breast cancer management [24,25,26,27,28,29,30,31,32,33,34,35,36,37,38,39,40,41,42,43,44,45,46,47]. This raises the possibility that other indirect interactions, namely, with cytostatic metabolites, may add on to or potentiate the effectiveness of chemotherapy agents used in the management of breast cancer, and we set out to investigate this possibility in a cell model of breast cancer. 

## 2. Results

### 2.1. General Consideratons

**Figure 1:**
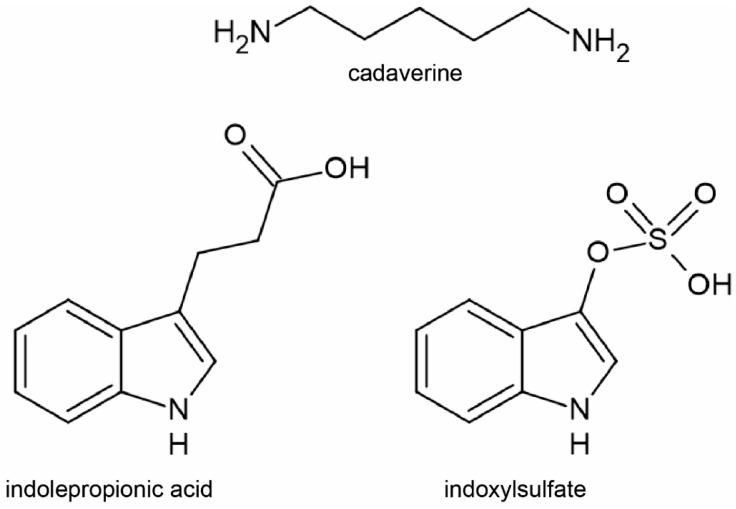
Chemical structure of bacterial metabolites used in the current study.

**Figure 2:**
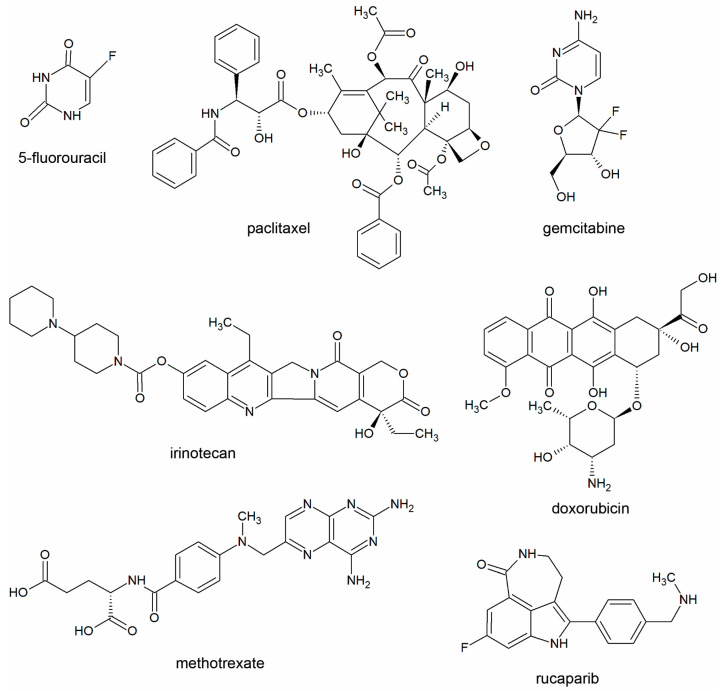
Structure of chemotherapy drugs used in the current study.

For the studies, we selected three well-characterized cytostatic bacterial metabolites, cadaverine (CAD), indolepropionic acid (IPA) and indoxylsulfate (IS), that were applied in concentrations corresponding to the top of the serum reference concentration of these metabolites, as follows: CAD: 0.8 µM [48,49], IPA: 1 µM [50,51,52] and IS: 4 µM [53]. The structure of these metabolites is shown in Figure 1. We investigated doxorubicin (DOX), gemcitabine (GEM), irinotecan (IRI), methotrexate (MTX), rucaparib (RUCA), 5-fluorouracil (5FU) and paclitaxel (PAC), all applied in a serial dilution series as indicated on the corresponding figures (similar to [54]). The structure of the chemotherapy agents is shown in Figure 2.

### 2.2. Bacterial Metabolites Do Not Interfere with Gemcitabine, Irinotecan, Methotrexate and Rucaparib Activity

**Figure 3:**
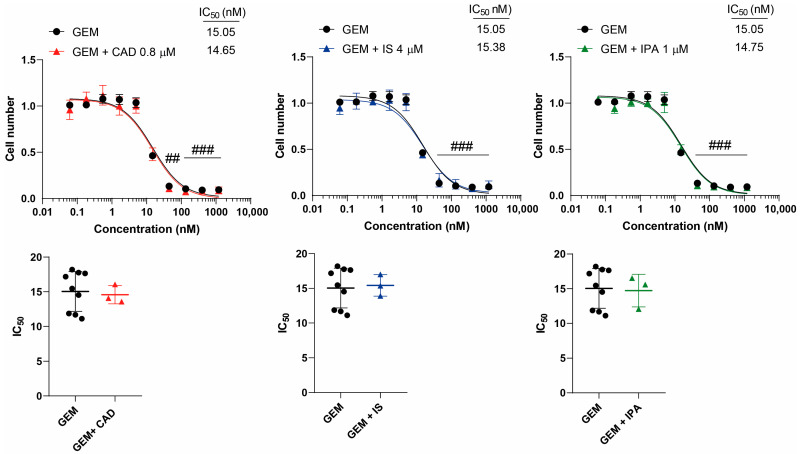
Cytostatic bacterial metabolites do not interfere with the cytostatic effect of gemcitabine. 4T1 cells were plated in 96-well plates (1500 cells/well). Cells were treated with gemcitabine alone or in combination with CAD (0.8 µM), IS (4 µM) or IPA (1 µM) for 48 h, and then cell numbers were determined by MTT assay. Data are presented as means ± SEM, from at least three biological replicates. Individual assays were measured in quadruplicate or in triplicate. Values were normalized to vehicle-treated cells (absorbance is equal to 1). Nonlinear regression (Graphpad “[Inhibitor] vs. response (three parameters)” utility) was performed on datasets to obtain IC_50_ values. Normality was determined for the inhibitory curves using the D’Agostino and Pearson normality test, while for the IC_50_ values the Shapiro–Wilk test was used. Dataset normality was achieved by the Box-Cox normalization method. Statistical difference between the inhibitory curves was determined using a two-way ANOVA test, and all data points were compared with each other (in Tukey post hoc tests). For the comparison of the IC_50_ values, a non-paired, two-sided t-test was applied. ## and ### indicate *p* < 0.01 and *p* < 0.001, respectively, in GEM-treated vs. vehicle-treated cells. Abbreviations: CAD—cadaverine; GEM—gemcitabine; IPA—indolepropionic acid; and IS—indoxylsulfate.

**Figure 4:**
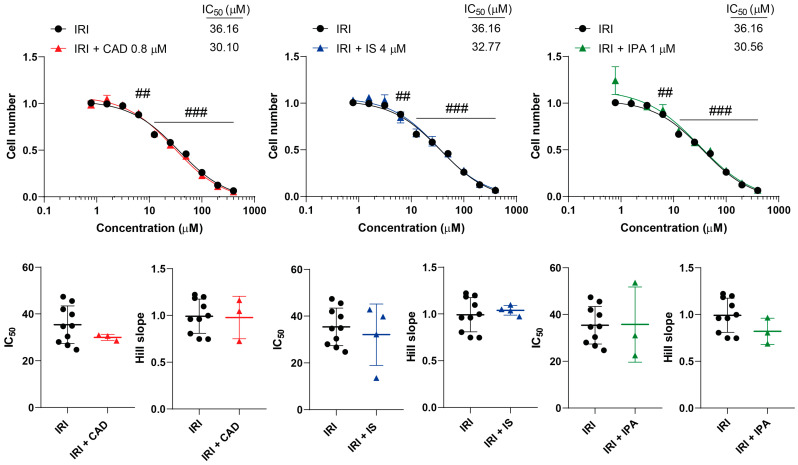
Cytostatic bacterial metabolites do not interfere with the cytostatic effect of irinotecan. 4T1 cells were plated in 96-well plates (1500 cells/well). Cells were treated with irinotecan alone or in combination with CAD (0.8 µM), IS (4 µM) or IPA (1 µM) for 48 h, and then cell numbers were determined by MTT assay. Data are presented as means ± SEM, from at least three biological replicates. Individual assays were measured in quadruplicate or in triplicate. Values were normalized to vehicle-treated cells (absorbance is equal to 1). Nonlinear regression (Graphpad “[Inhibitor] vs. response (four parameters)” utility) was performed on datasets to obtain IC_50_ and Hill slope values. Normality was determined for the inhibitory curves using the D’Agostino and Pearson normality test, while for the IC_50_ values and the Hill slope values the Shapiro–Wilk test was used. Statistical difference between the inhibitory curves was determined using a two-way ANOVA test, and all data points were compared with each other (in Tukey post hoc tests). For the comparison of the IC_50_ and Hill slope values, a non-paired, two-sided t-test was applied. ## and ### indicate *p* < 0.01 and *p* < 0.001, respectively, in IRI-treated vs. vehicle-treated cells. Abbreviations: CAD—cadaverine; IPA—indolepropionic acid; IRI—irinotecan; and IS—indoxylsulfate.

**Figure 5:**
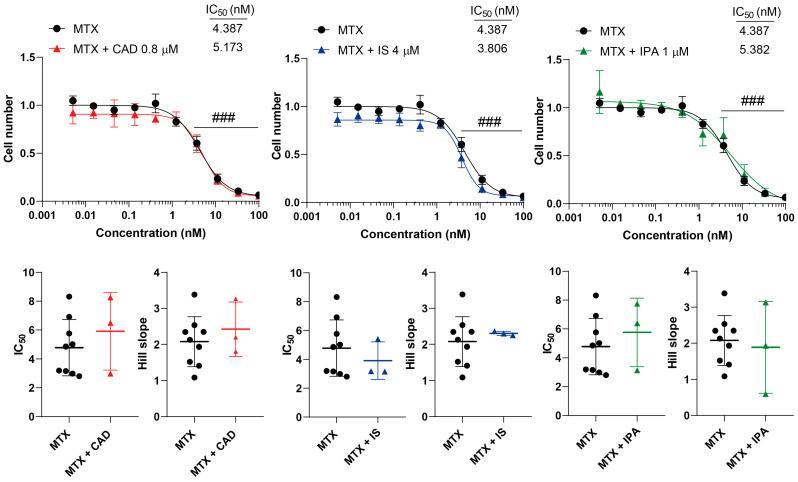
Cytostatic bacterial metabolites do not interfere with the cytostatic effect of methotrexate. 4T1 cells were plated in 96-well plates (1500 cells/well). Cells were treated with methotrexate alone or in combination with CAD (0.8 µM), IS (4 µM) or IPA (1 µM) for 48 h, and then cell numbers were determined by MTT assay. Data are presented as means ± SEM, from at least three biological replicates. Individual assays were measured in quadruplicate or in triplicate. Values were normalized to vehicle-treated cells (absorbance is equal to 1). Nonlinear regression (Graphpad “[Inhibitor] vs. response (four parameters)” utility) was performed on datasets to obtain IC_50_ and Hill slope values. Normality was determined for the inhibitory curves using the D’Agostino and Pearson normality test, while for the IC_50_ values and the Hill slope values the Shapiro–Wilk test was used. Statistical difference between the inhibitory curves was determined using a two-way ANOVA test, and all data points were compared with each other (in Tukey post hoc tests). For the comparison of the IC_50_ and Hill slope values, a non-paired, two-sided t-test was applied. ### indicates *p* < 0.001, for MTX-treated vs. vehicle-treated cells. Abbreviations: CAD—cadaverine; IPA—indolepropionic acid; IS—indoxylsulfate; and MTX—methotrexate.

**Figure 6:**
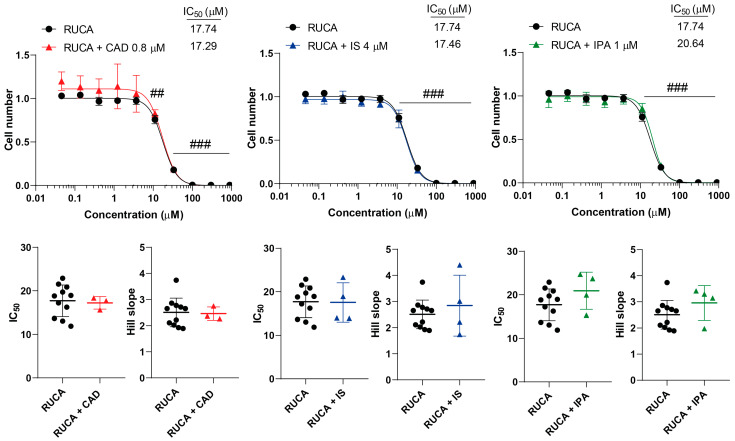
Cytostatic bacterial metabolites do not interfere with the cytostatic effect of rucaparib. 4T1 cells were plated in 96-well plates (1500 cells/well). Cells were treated with rucaparib alone or in combination with CAD (0.8 µM), IS (4 µM) or IPA (1 µM) for 48 h, and then cell numbers were determined by MTT assay. Data are presented as means ± SEM, from at least three biological replicates. Individual assays were measured in quadruplicate or in triplicate. Values were normalized to vehicle-treated cells (absorbance is equal to 1). Nonlinear regression (Graphpad “[Inhibitor] vs. response (four parameters)” utility) was performed on datasets to obtain IC_50_ and Hill slope values. Normality was determined for the inhibitory curves using the D’Agostino and Pearson normality test, while for the IC_50_ values and the Hill slope values the Shapiro–Wilk test was used. Statistical difference between the inhibitory curves was determined using a two-way ANOVA test, and all data points were compared with each other (in Tukey post hoc tests). For the comparison of the IC_50_ and Hill slope values, a non-paired, two-sided t-test was applied. ## and ### indicate *p* < 0.01 and *p* < 0.001, respectively, for RUCA-treated vs. vehicle-treated cells. Abbreviations: CAD—cadaverine; IPA—indolepropionic acid; IS—indoxylsulfate; and RUCA—rucaparib.

We tested the effects of CAD, IS and IPA on the inhibitory properties of GEM, IRI, MTX and RUCA in cell proliferation. None of the metabolites impacted the inhibitory activity of the chemotherapeutic agents, neither on the overall presentation of the inhibitory curves, nor on the kinetic readouts of the IC_50_ value or the Hill coefficient (Figure 3, Figure 4, Figure 5 and Figure 6, Table 1).

### 2.3. Bacterial Metabolites Interfere with 5-Fluorouracil

**Figure 7:**
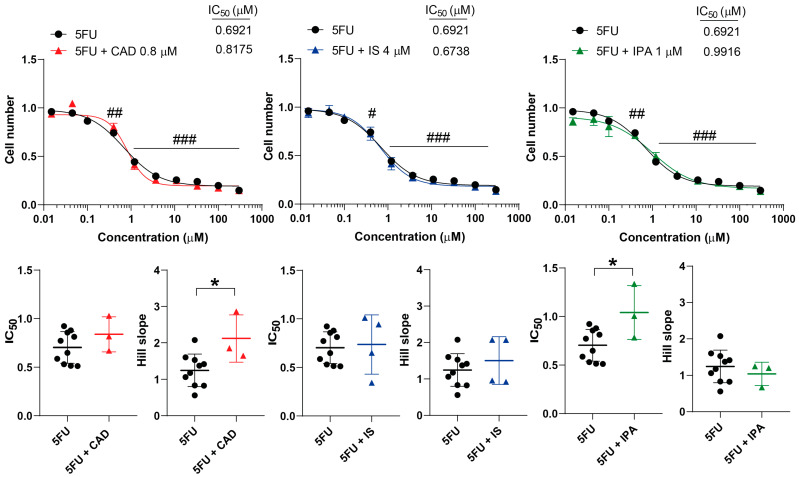
Cadaverine and indolepropionic acid interfere with the cytostatic effects of 5-fluorouracil. 4T1 cells were plated in 96-well plates (1500 cells/well). Cells were treated with 5-fluorouracil alone or in combination with CAD (0.8 µM), IS (4 µM) or IPA (1 µM) for 48 h, and then cell numbers were determined by MTT assay. Data are presented as means ± SEM, from at least three biological replicates. Individual assays were measured in quadruplicate or in triplicate. Values were normalized to vehicle-treated cells (absorbance is equal to 1). Nonlinear regression (Graphpad “[Inhibitor] vs. response (four parameters)” utility) was performed on datasets to obtain IC_50_ and Hill slope values. Normality was determined for the inhibitory curves using the D’Agostino and Pearson normality test, while for the IC_50_ values and the Hill slope values the Shapiro–Wilk test was used. To achieve normal distribution, datasets were log-normalized. Statistical difference between the inhibitory curves was determined using a two-way ANOVA test, and all data points were compared with each other (in Tukey post hoc tests). For the comparison of the IC_50_ and Hill slope values, a non-paired, two-sided t-test was applied. #, ## and ### indicate *p* < 0.05, *p* < 0.01 and *p* < 0.001, respectively, for 5FU-treated vs. vehicle-treated cells. * represents significance at *p* < 0.05 between the indicated groups. Abbreviations: CAD—cadaverine; IPA—indolepropionic acid; IS—indoxylsulfate; and 5FU—5-fluorouracil.

The three bacterial metabolites were tested together with 5FU, an antimetabolite chemotherapeutic agent. CAD increased the Hill coefficient, but did not change the IC_50_ value (Figure 7). Unfortunately, IPA increased the IC_50_ value of 5FU but did not affect the Hill coefficient (Figure 7). IS did not impact the kinetic properties of 5FU (Figure 7).

### 2.4. Bacterial Metabolites Interfere with Paclitaxel

**Figure 8:**
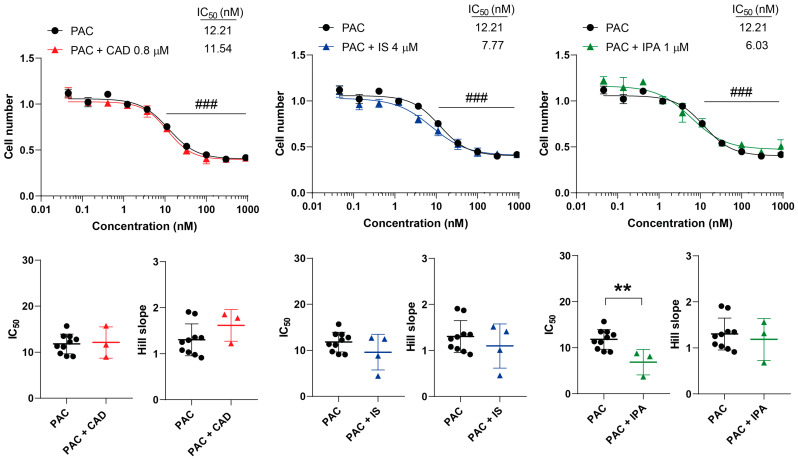
Indolepropionic acid improves the cytostatic effect of paclitaxel. 4T1 cells were plated in 96-well plates (1500 cells/well). Cells were treated with paclitaxel alone or in combination with CAD (0.8 µM), IS (4 µM) or IPA (1 µM) for 48 h, and then cell numbers were determined by MTT assay. Data are presented as means ± SEM, from at least three biological replicates. Individual assays were measured in quadruplicate or in triplicate. Values were normalized to vehicle-treated cells (absorbance is equal to 1). Nonlinear regression (Graphpad “[Inhibitor] vs. response (four parameters)” utility) was performed on datasets to obtain IC_50_ and Hill slope values. Normality was determined for the inhibitory curves using the D’Agostino and Pearson normality test, while for the IC_50_ values and the Hill slope values the Shapiro–Wilk test was used. Statistical difference between the inhibitory curves was performed using a two-way ANOVA test, and all data points were compared with each other (in Tukey post hoc tests). For the comparison of the IC_50_ and Hill slope values, a non-paired, two-sided t-test was applied. ### indicates *p* < 0.001 for5FU-treated vs. non-treated cells. ** represents significance at *p* < 0.01 between the indicated groups. Abbreviations: CAD—cadaverine; IPA—indolepropionic acid; IS—indoxylsulfate; and PAC—paclitaxel.

PAC is an antimicrotubule agent; it interferes with microtubule formation and movement during cell division. IPA decreased the IC_50_ value of PAC, while leaving the Hill coefficient unmodified (Figure 8). Furthermore, there was no interference with the kinetic properties of CAD and IS (Figure 8). 

### 2.5. Bacterial Metabolites Interfere with Doxorubicin

**Figure 9:**
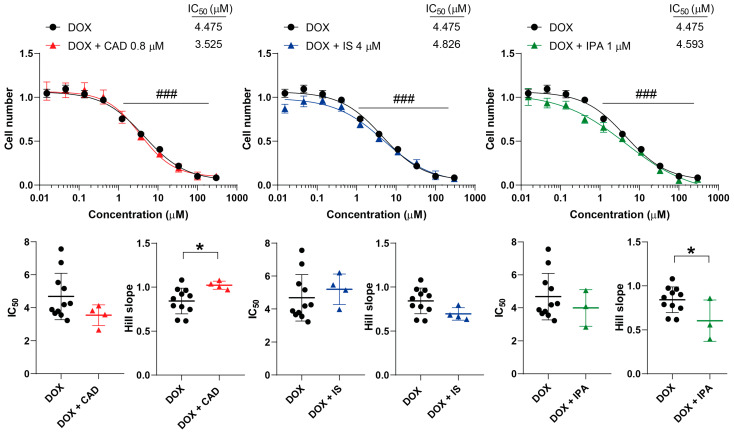
Cadaverine and indolepropionic acid interfere with the cytostatic effect of doxorubicin. 4T1 cells were plated in 96-well plates (1500 cells/well). Cells were treated with doxorubicin alone or in combination with CAD (0.8 µM), IS (4 µM) or IPA (1 µM) for 48 h, and then cell numbers were determined by MTT assay. Data are presented as means ± SEM, from at least three biological replicates. Individual assays were measured in quadruplicate or in triplicate. Values were normalized to vehicle-treated cells (absorbance is equal to 1). Nonlinear regression (Graphpad “[Inhibitor] vs. response (four parameters)” utility) was performed on datasets to obtain IC_50_ and Hill slope values. Normality was determined for the inhibitory curves using the D’Agostino and Pearson normality test, while for the IC_50_ values and the Hill slope values the Shapiro–Wilk test was used. Statistical difference between the inhibitory curves was determined using a two-way ANOVA test, and all data points were compared with each other (in Tukey post hoc tests). For the comparison of the IC_50_ and Hill slope values, a non-paired, two-sided t-test was applied. ### indicates *p* < 0.001 for DOX-treated vs. vehicle-treated cells. * represents significance at *p* < 0.05 between the indicated groups. Abbreviations: CAD—cadaverine; DOX—doxorubicin; IPA—indolepropionic acid; and IS—indoxylsulfate.

DOX is an anthracycline antibiotic that intercalates into DNA and disrupts topoisomerase II-mediated DNA repair and induces ROS production, contributing to cancer cell apoptosis [45]. CAD increased while IPA decreased the Hill coefficient (Figure 9). IS did not impact the kinetic properties of DOX (Figure 9).

## 3. Discussion

Chemotherapy plays a pivotal role in the management of breast cancer. Chemotherapy regimens are built on anthracyclines, cyclophosphamides, taxanes, antimetabolites (5-fluorouracil, gemcitabine and capecitabine), navelbine [55], targeted therapeutic agents as trastuzumab, pertuzumab and trastuzumab-emtansine, lapatinib [56], endocrine therapy, including selective estrogen receptor modulators (SERMs), aromatase inhibitors and GNRH-analogs [56] and novel therapeutic agents as PARP inhibitors [57,58] or CDK4/6 (cyclin-dependent kinases) inhibitors [59]. In this study, we assessed the inhibitors that can be utilized in cell-based model systems, as their action does not require activation in the liver, interaction with the immune system (as for humanized antibodies) or systemic endocrine loops (e.g., SERMs). 

Hereby, we investigated whether there is an interaction between cytostatic bacterial metabolites and the above-mentioned chemotherapy agents. Numerous bacterial metabolites with bioactivity in neoplasias were identified, the majority of which have cytostatic properties [2,3,4,5,6,7,8,9,10,11,12,13]. The production of these metabolites decline in breast cancer patients; nevertheless, administration of minute quantities of these metabolites reduces the mitotic rate and the metastatic potential of the primary tumor [3,4,5,6]. Such metabolites exert their effects though multifaceted processes, at the root of which an anti-Warburg-type metabolic rearrangement and the induction of mild oxidative stress can be found. These elementary events inhibit the epithelial–mesenchymal transition and lower the proportions of cancer stem cells, concluding in cytostasis and a reduced metastatic and recurrence potential [2].

Multiple chemotherapy agents have been shown to modulate the composition of the microbiome [60,61]; bacterial metabolism of chemotherapy agents has also been evidenced [31,33,34,35,36,37,38,39], and the efficiency of humanized antibodies [62] has also been linked to compositional changes in the microbiome. These observations have raised the possibility that bacterial metabolites may interfere with the cytostatic or cytotoxic effects of chemotherapy agents. In the current study, we identified that CAD and IPA do interfere with PAC, 5FU and DOX. Other metabolites with similar properties have already been identified. Urolithin A was shown to mitigate drug resistance to 5FU through the FOXO3-FOXM1 pathway in colorectal cancer [40]. Indole-3-acetic acid increased the efficacy of chemotherapy in murine models and in humans [63]. Importantly, shifting the redox status of cancer cells towards a more oxidative phenotype played a key role in the beneficial effects of indole-3-acetic acid [63]. Another study reported that bacterial metabolites can impact Warburg metabolism and, hence, interfere with chemoradiotherapy [64]. Finally, ursodeoxycholic acid was shown to potentiate the activity of sorafenib on hepatocellular carcinoma cells [65]. These observations align with the reports showing that the metabolites applied in the current study also interfere with these processes [3,4,5,6,66], which are possible explanations for the effects observed. It is also of note that the interference of bacterial metabolites with chemotherapy agents in breast cancer cell models, presented in this study, contrasts to our negative observations of the interactions between bile acids and chemotherapy agents in pancreatic adenocarcinoma cells [67,68]. The bioactivity of certain microbiome-derived metabolites is extensively reviewed in the following papers: [41,42,43,44,45,46]. 

Protective bacterial species and bacterial metabolites were identified against the side effects of chemoradiotherapy [69,70]. It is of note that the bacterial metabolites assessed in this study did not display toxicity towards non-transformed cells as in previous studies and can be applied in low concentrations [4,5,6], pointing towards the likeliness of a safe application of these metabolites in therapeutic settings.

CAD increased the Hill coefficient of 5FU and DOX, suggesting a more collaborative binding or effect of the drug molecules [71], while IPA decreased the Hill coefficient of DOX, suggesting a less collaborative binding or effect. It is difficult to explain the mechanism through which these metabolites can affect the binding or effectiveness of the chemotherapy drugs. In line with this observation, these findings have an unpredictable pharmacological relevance. 

In contrast, IPA increased the IC_50_ value of 5FU, suggesting a lower efficiency that has negative pharmacological and, likely, clinical consequences. Meanwhile, in the case of PAC the IC_50_ value was halved in the presence of IPA, making this combination potentially advantageous. These findings also suggest that IPA may have adverse effects when PAC+5FU combinations are applied.

IPA is a bacterial metabolite that is the synthesized from tryptophan through deamination by tryptophanase (TnaA) [4,52]. A significant portion of tryptophan (4–6%) undergoes bacterial catabolism [72]. Multiple studies have shown that disturbances to indole/tryptophan metabolism correlates with survival in breast cancer ([5,6,73,74], reviewed in [5]). Our observations extend these studies by adding that higher IPA levels may support PAC responsiveness.

## 4. Materials and Methods

### 4.1. Chemicals

Bacterial metabolites (Cadaverine-CAD, cat # C8561; Indoxylsulfate-IS, cat # 13875; and Indolepropionic acid-IPA, cat # 220027) were purchased from Sigma-Aldrich (St. Louis, MI, USA). All metabolites were dissolved in dimethyl-sulfoxide (DMSO) at a stock concentration of 100 mM. CAD was used at concentrations of 0.8 μM, IS at 4 μM and IPA at 1 μM, corresponding to normal human serum concentrations of these metabolites [48,49,50,51,52,53]. 

Chemotherapy drugs, Irinotecan (IRI, cat # I1406), 5-fluorouracil (5FU, cat # F6627), Methotrexate (MTX, cat # PHR1396), Rucaparib (RUCA, cat # PZ0036) and Gemcitabine (GEM, cat # G6423) were from Sigma-Aldrich. The drugs IRI, 5-FU, MTX and RUCA were dissolved in DMSO at a stock concentration of 100 mM; GEM was dissolved in water at a stock concentration of 100 mM. Liposomal Encapsuled Doxorubicin (DOX-NP, cat # 300112) was purchased from Avanti Polar Lipids (Alabaster, AL, USA) and a stock solution of 50 mM was prepared. Paclitaxel (PAC, cat # A0451335) was from Thermo Fisher Scientific (Waltham, MA, USA) and 50 mM stock solution was prepared in DMSO. 

Chemotherapy compounds were used at different concentrations as indicated in the figures.

### 4.2. Cell Line

The 4T1 breast cancer cell line was obtained from the American Type Culture Collection. Cells were cultured in RPMI-1640 medium (Sigma-Aldrich, cat # R5886) containing 10% fetal bovine serum (FBS), 1% penicillin/streptomycin, 2 mM L-glutamine and 1% pyruvate at 37 °C in a humidified incubator with 5% CO_2_. Cells were regularly checked for Mycoplasma contamination.

### 4.3. MTT Assay

4T1 cells were plated in 96-well plates (1.5 × 10^3^ cell/well). On the next day, cells were treated with chemotherapy agents alone or in combination with bacterial metabolites for 48 h. After treatments, cell numbers were determined using an MTT (3-(4,5-dimethylthiazol-2-yl)-2,5-diphenyltetrazolium bromide) assay. Briefly, cells were treated with MTT solution (0.5 mg/mL) and incubated at 37 °C for 90 min. Then, the culture medium was discarded and the formazan crystals were dissolved in DMSO. The absorbance was measured on a plate reader (Thermo Labsystems Multiskan MS, Walthman, MA, USA) at 540 nm. In the calculations, the absorbance values for the vehicle-treated cells were considered 1, and all treatment were expressed relative to 1.

### 4.4. Statistical Analysis

Each analysis was performed using GraphPad Prism Version 8.0.1 (244) software. Experiments were repeated at least three times and results are presented as mean ± SEM values. Normal distribution of the values was tested using the D’Agostino and Pearson normality test. Where appropriate, values were log-normalized or normalized using the Box-Cox normalization method [75]. Nonlinear regression was performed using the GraphPad “[Inhibitor] vs. response—Variable slope (four parameters)” utility, from which IC_50_ and Hill slope values were obtained unless otherwise stated. A two-way analysis of variance test followed by Tukey’s honestly significant post hoc test were used for multiple comparisons.

## 5. Conclusions

The oncobiome was shown to modulate the efficacy or even limit the availability of chemotherapy agents. In this study, we showed that, in contrast to previous negative findings in pancreatic adenocarcinoma models, IPA and CAD modulated the cytostatic activity of 5FU, PAC and DOX. CAD and IPA modulated the Hill coefficient of 5FU and DOX, which has unpredictable pharmacological significance. IPA increased the IC_50_ value of 5FU, which is a disadvantageous interaction. Importantly, IPA decreased the IC_50_ values of PAC, which is a beneficial interaction, as PAC concentrations can be decreased in combination with a low concentration of a non-toxic compound, which may limit the side effects of PAC.

## Figures and Tables

**Table 1:** The kinetic values of the metabolite–chemotherapy agent combinations. * and ** represent significance at *p* < 0.05 and *p* < 0.01 between the indicated group and the corresponding control.

Chemotherapeutic Agent	Metabolite	IC_50_ (±SD)	Hill Coefficient (±SD)
Gemcitabine	-	15.05 (±2.87)	-
	Cadaverine	14.65 (±1.31)	-
	Indoxylsulfate	15.38 (±1.55)	-
	Indolepropionic acid	14.75 (±2.35)	-
Irinotecan	-	36.16 (±8.03)	0.93 (±0.18)
	Cadaverine	30.10 (±1.30)	0.97 (±0.22)
	Indoxylsulfate	32.77 (±13.13)	1.03 (±0.05)
	Indolepropionic acid	31.56 (±16.07)	0.81(±0.14)
Methotrexate	-	4.38 (±1.94)	2.07 (±0.69)
	Cadaverine	5.17 (±2.68)	2.42 (±0.75)
	Indoxylsulfate	3.80 (±1.29)	2.30 (±0.05)
	Indolepropionic acid	5.38 (±2.37)	1.88 (±1.27)
Rucaparib	-	17.74 (±3.63)	2.50 (±0.54)
	Cadaverine	17.29 (±1.43)	2.46 (±0.25)
	Indoxylsulfate	17.46 (±4.52)	2.84 (±1.16)
	Indolepropionic acid	20.64 (±4.26)	2.95 (±0.66)
5-fluorouracil	-	0.69 (±0.16)	1.24 (±0.44)
	Cadaverine	0.81 (±0.17)	2.12 (±0.64) *
	Indoxylsulfate	0.67 (±0.30)	1.50 (±0.65)
	Indolepropionic acid	0.99 (±0.27) *	1.04 (±0.32)
Paclitaxel	-	12.21 (±2.12)	1.30 (±0.34)
	Cadaverine	11.54 (±3.38)	1.61 (±0.34)
	Indoxylsulfate	7.77 (±3.86)	1.09 (±0.47)
	Indolepropionic acid	6.03 (±2.76) **	1.18 (±0.45)
Doxorubicin	-	4.47 (±1.40)	0.84 (±0.14)
	Cadaverine	3.52 (±0.62)	1.02 (±0.04) *
	Indoxylsulfate	4.82 (±0.92)	0.69 (±0.06)
	Indolepropionic acid	4.59 (±1.10)	0.60 (±0.23) *

## Data Availability

Primary data of the present manuscript can be found at https://figshare.com/s/6ecb8c6bd8b87284ae3c (DOI: 10.6084/m9.figshare.25678635) (accessed on 29 April 2024).
